# Withdrawal: The role of CXCR7/RDC1 as a chemokine receptor for CXCL12/SDF-1 in prostate cancer

**DOI:** 10.1016/j.jbc.2022.102126

**Published:** 2022-06-20

**Authors:** Jianhua Wang, Yusuke Shiozawa, Jincheng Wang, Yu Wang, Younghun Jung, Kenneth J. Pienta, Rohit Mehra, Robert Loberg, Russell S. Taichman

This article has been withdrawn by the authors to correct the scientific record, except for Jianhua Wang who could not be contacted. The journal analysis and the authors conclude that in Figure 5C, the C4-2B^CXCR7^ panel has been rotated and reused as the C4-2B^siControl^ panel. Due to primary data for C4-2B^siControl^ panel not supporting the original conclusions, the authors wish to withdraw this article.

